# Cardiovascular and metabolic status in patients with primary hyperparathyroidism: a single-center experience

**DOI:** 10.3389/fendo.2023.1266728

**Published:** 2023-09-29

**Authors:** Ekaterina A. Dobreva, Anna M. Gorbacheva, Ekaterina E. Bibik, Anna K. Eremkina, Alina R. Elfimova, Rustam Kh. Salimkhanov, Elena V. Kovaleva, Irina S. Maganeva, Natalia G. Mokrysheva

**Affiliations:** ^1^ Department of Parathyroid Glands Pathology, Endocrinology Research Centre, Moscow, Russia; ^2^ Administration, Endocrinology Research Centre, Moscow, Russia

**Keywords:** primary hyperparathyroidism, cardiovascular diseases, arterial hypertension, diabetes mellitus, dyslipidemia, metabolic diseases

## Abstract

**Introduction:**

Cardiovascular diseases (CVD) and metabolic disorders (MD) have retained leading positions in the structure of morbidity and mortality for many years. Primary hyperparathyroidism (PHPT) is also associated with a greater incidence of CVD and MD. The aim of the present study was to describe the prevalence and structure of CVD and MD in hospitalized patients with PHPT and to search for possible associations between these pathologies.

**Methods:**

838 patients with a verified PHPT were included in the study. The studied cohort was divided into 2 groups according to their age at the time of admission: patients aged 18 to 49 years (group A, n = 150); patients aged 50 years and older (group B, n = 688).

**Results:**

There were no significant differences between two groups in parameters of calcium-phosphorus metabolism. Obesity was diagnosed in 24.2% of patients in group A and in 35.9% in group B. Type 2 diabetes mellitus was more common in older patients (14.4% in group B vs. 2.6% in group A). Arterial hypertension, ischemic heart disease, chronic heart failure and brachiocephalic arteries atherosclerosis were more frequent in older patients, occurring in 79.1%, 10.8%, 8.4%, and 84% of cases respectively. The cutoff points that increased the risk of CVD detection turned out to be age above 56 years, eGFR below 92 ml/min/1.73m2, BMI above 28.3 kg/m2.

**Discussion:**

The present study demonstrated a high incidence of some CVD, as well as disorders of lipid, carbohydrate and purine metabolism in patients with PHPT.

## 1 Introduction

Cardiovascular diseases (CVD) and metabolic disorders (MD) have consistently held top positions in causing illness and death. Furthermore, disruptions in calcium-phosphate metabolism, such as primary hyperparathyroidism (PHPT), have also shown to be linked to increased occurrences of both CVD (1) and MD (2, 3).

PHPT is one of the most common endocrine disorders and primarily affects postmenopausal women who are at increased risk of CVD. Traditionally, there has been a significant focus on the “classic” complications of PHPT, such as osteoporosis and nephrocalcinosis/nephrolithiasis (4). Nevertheless, in recent years, there has been a growing interest in the “non-classical manifestations”, such as CVD and metabolic syndrome, due to the increasing prevalence of asymptomatic PHPT (5, 6). PHPT is linked to a greater risk of mortality than the general population, independent of current calcium levels. Moreover, even asymptomatic PHPT-patients have an increased risk of cardiovascular death (7). Nonetheless, the occurrence of CVD and MD in patients with PHPT, along with their underlying connections, remain uncertain.

The purpose of this study is to report the frequency and structure of CVD and MD in hospitalized patients with PHPT and to explore potential connections between these conditions.

## 2 Materials and methods

### 2.1 Study design

A single-center, single-stage comparative study was performed. The sample included patients who were admitted to the Department of Parathyroid Gland Pathology and Mineral Metabolism Disorders of the Endocrinology Research Centre, Moscow from January 2017 to December 2022. The continuous sampling method was used.

The inclusion criteria were PHPT verified in accordance with current clinical guidelines (4, 8); age ≥18 years; presence of the patient’s signed informed and voluntary consent for inpatient examination and treatment.

The exclusion criterion was the patient’s refusal to continue the examination and treatment.

Patients with genetically verified syndromes of multiple endocrine neoplasia or with their phenocopies were excluded from the study, because concomitant hormonal disorders could significantly affect the severity of cardiovascular pathology, carbohydrate, lipid and purine metabolism. Patients with diabetes, given its high prevalence in the population, were not excluded from the study. Laboratory and instrumental data were analyzed, as well as information from cardiologists’ records. Patients’ body weight was measured using electronic floor-standing medical scales (“VEM-150”, Massa-K, Russia). Patients’ height was measured using a medical stadiometer (“R-Ss-MSK MSK-233”, Medstalkonstruktsia, Russia). Obesity was diagnosed according to World Health Organization criteria and body mass index (BMI).

Patients underwent several laboratory tests. ARCHITECT c8000 chemistry analyzer (Abbott, USA) was used to establish concentrations of serum total and ionized calcium (Ca), albumin, alkaline phosphatase (ALP), phosphorus, creatinine, cholesterol of low-density lipoproteins (LDL), high-density lipoproteins (HDL), triglycerides, total cholesterol (TC), glucose, serum uric acid, and daily calciuria values. D10 tester (BioRad, USA) was used to determine glycated hemoglobin (HbA1c) by high-performance liquid chromatography. Cobas 6000 electrochemiluminescence analyzer (Roche, Germany) was used to estimate the serum parathyroid hormone (PTH), osteocalcin, C-terminal telopeptide of type 1 collagen (CTX) concentrations. Albumin corrected calcium was calculated using the formula total calcium (mmol/l) = calcemia (mmol/l) + 0.02×(40 — albumin level (g/l)). Estimated glomerular filtration rate (eGFR) was established using the CKD-EPI 2009 formula. Serum 25(OH) vitamin D concentrations were assessed in patients’ local laboratories because it is not included in the standards of in-patient examination of PHPT; we collected information on this parameter from their medical records.

Patients underwent examination for bone and visceral complications of PHPT, including dual-energy X-ray absorptiometry (DXA) scans of lumbar spine, femur and radius (Lunar iDXA, GE Healthcare, Japan or Discovery, Hologic, USA); X-ray of the thoracic and lumbar spine (X-ray diagnostic system Optima RF420, GE Healthcare, Japan); kidney ultrasound (Aplio 500, Toshiba, Japan) and|or kidney CT-scan (Optima CT660, GE, USA); esophagogastroduodenoscopy (Olympus GIF-XP 150N, Olympus Corporation, Japan).

All patients were screened for CVD, including electrocardiography (ECG, PageWriter TC70, Philips, The Netherlands), for all patients; echocardiography (VIVID E95, “GE Healthcare”, Japan) - if indicated; daily ECG monitoring and daily BP monitoring (Medilog DARWIN Professional, “Schiller”, Switzerland) - if indicated; ultrasound of brachiocephalic arteries (BCA) and arteries in lower extremities (Aplio 500, “Toshiba”, Japan) - if indicated.

Presence or absence of CVD in all patients was established during an examination by a cardiologist. In addition, patients’ prescription histories were collected; the medications that patients received on admission and at discharge were taken into account. If patients had arterial hypertension (AH), they were discharged when they reached predominantly normotonic or blood pressure (BP) values close to target during the day.

### 2.2 Statistical analysis

Statistical data analysis was performed using the Statistica software package v.13.3 (TIBCO Software Inc., USA). Descriptive statistics of quantitative variables are represented by the median and the first and third quartiles as Me [Q1; Q3]. Qualitative indicators are presented in absolute (n) and relative (%) frequencies. Confidential intervals (CI 95%) for frequencies were calculated by the Klopper–Pearson method. A comparative analysis of two independent groups (subgroups) studied by quantitative characteristics was carried out using the Mann-Whitney U test, three or more groups – using the Kruskel–Wallis H test. Comparison of independent groups by qualitative characteristics was carried out using the Chi-squared test (χ2), with a value of at least one expected frequency less than 5, the Yates correction was applied. Spearman’s rank correlation was used for analysis of associations between quantitative parameters. A p-value less than 0.05 is considered to be statistically significant when testing statistical hypotheses. The Bonferroni correction was applied to counteract the multiple comparisons problem. The p-values in the range between the calculated p0 and 0.05 were interpreted as a statistical trend.

## 3 Results

### 3.1 Sample description

A total of 838 patients with a verified PHPT were included in the study, among them 775 women (92.5%) and 63 men (7.5%). The median age at the admission was 59 years [51; 66], 53 [38; 66] years for men, and 60 [52; 66] years for women. The median duration of PHPT was 2 [1;3] years, whereas the maximum disease duration was 22 years.

The studied cohort was divided into 2 groups according to their age at the time of admission: patients aged 18 to 49 years (group A, n = 150); patients aged 50 years and older (group B, n = 688). Patients from group A had predictably higher eGFR (and consequently higher calciuria and lower phosphatemia), and a lower incidence of bone complications. There were no differences in the incidence of visceral complications between the groups. The general characteristics of the studied groups are presented in **Table 1**.

**Table 1:** State of mineral metabolism in studied groups.

Parameter	Reference range	Group А (n = 150)		Group В (n = 688)		р
		N	Ме [Q1; Q3], n (%)	N	Ме [Q1; Q3], n (%)	
Са total.. mmol/l	2.15–2.55	150	2.8 [2.7; 3.0]	688	2.8 [2.7; 2.9]	0.034^1^
Са ionized. mmol/l	1.03–1.29	75	1.3 [1.2; 1.4]	411	1.3 [1.2; 1.4]	0.236^1^
Са corrected. mmol/l	2.15–2.55	150	2.7 [2.6; 2.9]	683	2.7 [2.6; 2.8]	0.079^1^
Phosphorus. mmol/l	0.74–1.52	145	0.8 [0.7; 0.9]	671	0.9 [0.8; 1.0]	**<0.001^1^**
Creatinine. mmol/l	50–98	148	67 [61;73]	687	69.2 [63.3; 78.4]	**0.001^1^**
eGFR by CKD–EPI. ml/min/1.73m^2^	–	148	101 [92; 110]	686	82 [71; 91]	**<0.001^1^**
25 (ОН) D. ng/ml	30–100	74	21 [12; 28]	334	24 [17; 34]	0.004^1^
PTH. pg/ml	15–65	150	132.3 [107; 257]	687	132 [99.9; 209.5]	0.117^1^
Calciuria. mmol/24h	2.5–8	145	9.3 [7.3; 11.3]	659	7.7 [5.2; 10.5]	**<0.001^1^**
ALP. u/l	40–150	136	82 [61; 111.5]	609	88 [68; 115]	0.138^1^
Osteocalcin. ng/ml	11–43	111	49.5 [32.3; 96.3]	496	44 [28.3; 68.3]	0.003^1^
СТХ. ng/ml	0.3–0.57	109	1 [0.6; 1.7]	494	0.9 [0.5; 1.3]	**0.002^1^**
Bone complications		150	50 (33.3%)	687	478 (69.5%)	**< 0.001^2^**
♦ Among them: BMD < 2.0 SD (Z-score) or < 2.5 SD (T-score)		149	48 (32%)	686	467 (68%)	**<0.001^2^**
♦ Among them: vertebral fractures		150	6 (4%)	686	103 (15%)	**<0.001^2^**
♦ Among them: low-energy non-vertebral fractures		149	11 (7.4%)	684	85 (12.4%)	0.080^2^
♦ Among them: clinical signs of fibrocystic osteitis		150	9 (6%)	686	16 (2.3%)	0.010^2^
Visceral complications		125	95 (76.0%)	530	436 (82.3%)	0.107^2^

^1^ – Mann-Whitney test, ^2^ – χ^2^ test; р_0 = _0.05/20 = 0.002 (Bonferroni correction).

Visceral complications include kidney and gastrointestinal manifestations of PHPT.

Bold values mean statistic significance of the p-value.

### 3.2 Lipid and purine metabolism

Obesity was diagnosed in 24.2% of patients in group A and in 35.9% in group B. BMI in group A was significantly lower. Groups were comparable in other parameters of lipid and purine metabolism (**Table 2**).

**Table 2:** Lipid and purine metabolism in the studied groups.

Parameter	Reference range	Group А (n = 150)		Group В (n = 688)		р
		N	Ме [Q1; Q3], n (%)	N	Ме [Q1; Q3], n (%)	
Obesity:	–	149	36 (24.2%)	685	246 (35.9%)	0.006^2^
BMI, kg/m^2^	20–25	149	26 [22; 29]	686	28 [25; 32]	**< 0.001** ^1^
TC, mmol/l	3.3–5.2	148	5.1 [4.6; 5.9]	679	5.5 [4.7; 6.4]	0.006^1^
LDL, mmol/l	1.1–3	146	3.3 [2.6; 4.0]	676	3.5 [2.7; 4.3]	0.040^1^
HDL, mmol/l	1.15–2.6	142	1.4 [1.1; 1.6]	635	1.4 [1.1; 1.6]	0.826^1^
Triglycerides, mmol/l	0.1–1.7	145	1.2 [0.8; 1.8]	667	1.3 [1.0; 1.8]	0.032^1^
Uric acid, mcmol/l	142–339	110	313.3 [278.5; 364.4]	552	336.7 [288.2; 407.7]	0.022^1^

^1^ – Mann-Whitney test; ^2^ – χ^2^ test; р_0 = _0.05/13 = 0.004 (Bonferroni correction).

Bold values mean statistic significance of the p-value.

### 3.3 Сarbohydrate metabolism

As expected, type 2 diabetes mellitus was more common in older patients (14.4% in group B vs. 2.6% in group A). There was a trend towards a higher incidence of the impaired glucose tolerance/impaired fasting glycemia (IGT/IFG) in group B. Among patients without carbohydrate metabolism disorders, fasting glycemia and HbA1c were also higher in group B. Detailed information is provided in **Table 3**.

**Table 3:** Carbohydrate metabolism in the studied groups.

Indicator	Reference range	Group А (n = 150)		Group В (n = 688)			р	
		N	Ме [Q1; Q3], n (%)	N	Ме [Q1; Q3], n (%)			
Type 2 diabetes mellitus (including previously diagnosed)	–	150	4 (2.6%)	686	99 (14.4%)		**< 0.001** ^2^
IGT/IFG (including previously diagnosed)	–	150	3 (2.0%)	686	50 (7.3%)		0.016^2^
Among patients without diabetes, IGT/IFG								
Fasting glucose, mmol/l	3.1–6.1	136	4.9 [4.6; 5.3]	507	5.2 [4.9; 5.5]		**< 0.001** ^1^	
HbA1c,%	4–6	19	5.3 [5.0; 5.4]	41	5.6 [5.3; 5.8]		**0.002** ^1^	

^1^ – Mann-Whitney test; ^2^ – χ^2^ test; р_0 = _0.05/4 = 0.012 (Bonferroni correction).

Bold values mean statistic significance of the p-value.

### 3.4 Cardiovascular diseases in the studied group

Detailed CVD structure of the studied population is provided in **Table 4**. As expected, BCA atherosclerosis, AH, ischemic heart disease (IHD), and chronic heart failure (CHF) were more frequent in older patients, occurring in 84%, 79.1%, 10.8%, and 8.4% of cases respectively. We also compared both groups for the main parameters of ECG and echocardiography. Patients in the group B had longer corrected QT intervals (410,9 [396,7; 425,8] ms vs. 405,2 [389,7; 418,6] ms), as well as greater left ventricle (LV) thickness (10 [9; 11] mm vs. 9 [8; 9] mm), higher interventricular septal thickness (IST, 10 [9; 11] mm vs. 9 [8; 9,3] mm) and LV mass index (86 [75; 98] g/m^2^ vs. 74 [65; 85] g/m^2^), p<0,001 for all.

**Table 4:** Cardiovascular diseases and conditions in the studied groups.

Parameter	Group А (n = 150)		Group В (n = 688)		р
	*N*	Ме [Q1; Q3], n (%)	*N*	Ме [Q1; Q3], n (%)	
AH	149	43 (28.9%)	688	544 (79.1%)	**< 0.001** ^1^
IHD	148	1 (0.7%)	684	74 (10.8%)	**< 0.001** ^1^
CHF	150	0 (0.0%)	688	58 (8.4%)	**< 0.001** ^1^
Cardiac rhythm disorders	150	14 (9.3%)	686	81 (11.8%)	0.387^1^
Conduction disorders	148	14 (9.5%)	685	120 (17.5%)	0.016^1^
BCA atherosclerosis	9	1 (11.1%)	100	84 (84%)	**< 0.001** ^1^
Lower extremity arteries atherosclerosis	10	1 (10.0%)	39	19 (48.7%)	0.263^1^

^1^ – χ^2^ test; р_0 = _0.05/26 = 0.002 (Bonferroni correction).

Bold values mean statistic significance of the p-value.

### 3.5 Associations between mineral metabolism and cardiovascular diseases

Also patients with CVDs (AH, CHF, heart rhythm disorders, conduction disorders, BCA atherosclerosis, atherosclerosis of lower extremity arteries, IHD) and those without were compared separately for mineral metabolism and demographic parameters. The groups significantly differed in age, eGFR, and BMI, HDL and uric acid levels. Differences at the statistical trends level were obtained for ALP and triglycerides concentration, as well as for incidence of bone complications. Results are presented in **Table 5**.

**Table 5:** Comparative analysis of patients with and without CVDs.

	*Patients with CVDs*		*Patients without CVDs*		*р*
	N	Me [Q1; Q3] or n (%)	N	Me [Q1; Q3]	
Male patients	612	48 (8%)	226	15 (7%)	0.660^2^
Age, years	612	62 [55;69]	226	51 [39;58]	<0.001^1^
Ca total, mmol/l	612	2.76 [2.65;2.92]	226	2.75 [2.65;2.91]	0.311^1^
Са ionized, mmol/l	365	1.30[1.25;1.37]	122	1.29 [1.25;1.37]	0.926^1^
Са corrected, mmol/l	607	2.67 [2.57;2.83]	226	2.65 [2.56;2.81]	0.126^1^
Phosphorus, mmol/l	597	0.87 [0.77;0.96]	218	0.86 [0.75;0.97]	0.459^1^
eGFR, ml/min/1,73m^2^	610	70 [63;81]	224	93 [84;1.3]	<0.001^1^
25 (OH) D, ng/ml	302	24 [16;33]	108	22 [15;32]	0.285^1^
PTH, pg/ml	612	135 [104;216]	226	124 [98;205]	0.068^1^
BMI, kg/m2	612	28.8 [25.1;32.7]	226	25.7 [23.4;29.0]	<0.001^1^
ALP, U/l	552	90 [69;117]	193	79 [62;107]	0.003^1^
Osteocalcin, ng/ml	445	45.97 [28.86; 74.94]	162	43 [30;68]	0.985^1^
CTX, ng/ml	445	0.87 [0.51;1.36]	160	0.91 [0.57;1.37]	0.469^1^
Diabetes mellitus	611	94 (15%)	226	10 (4%)	<0.001^2^
Fasting glucose, mmol/l	564	5.27 [4.90;5.73]	209	5.06 [4.71;5.38]	<0.001^1^
Total cholesterol, mmol/l	604	5.47 [4.63;6.34]	223	5.46 [4.89;6.31]	0.442^1^
LDL, mmol/l	602	3.5 [2.6;4.3]	220	3.5 [2.8;4.3]	0.405^1^
HDL, mmol/l	568	1.34 [1.12;1.54]	209	1.47 [1.17;1.72]	<0.001^1^
Triglycerides, mmol/l	595	1.3 [0.96;1.8]	218	1.17 [0.87;1.65]	0.005^1^
Uric acid, mcmol/l	495	345 [294;416]	167	307 [270;345]	<0.001^1^
Bone complications	612	404 (66%)	226	124 (54%)	0.004^2^
Visceral complications	612	393 (64%)	226	138 (61%)	0.447^2^

^1^ – U-test; ^2^ – χ^2^ test with Yates correction; Bonferroni correction р_0 = _0.05/9 = 0.006.

**Figure 1:**
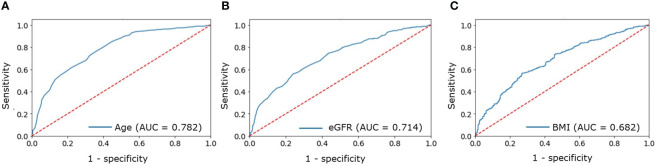
ROC analysis for: **(A)** — age; **(B)** — eGFR; **(C)** — BMI to determine increased probability of CVDs in patients with PHPT.

A ROC analysis was performed to identify cutoff points suggestive of CVD in a patient with PHPT. The cutoff points that increased the risk of CVD detection turned out to be:

- Age above 56 years (AUC = 0.782, 95% CI [0.751–0.812], **Figure 1А**);
- eGFR below 92 ml/min/1.73m^2^ (AUC = 0.714, 95% CI [0.674–0.753], **Figure 1B**);
- BMI above 28.3 kg/m^2^ (AUC = 0.682, 95% CI [0.645–0.719], **Figure 1C**).

The OR for the combination of all three factors was 4.11 (95% CI [2.99-5.79]).

In order to evaluate potential relationships between mineral metabolism parameters and state of carbohydrate, lipid, and purine metabolism, as well as incidence of CVD, the correlation analysis was performed. The analysis concerning lipid and purine metabolism was performed in patients with eGFR ≥60 mL/min/1.73m^2^.

We found weak positive correlations between values of Ca cor. and serum total cholesterol and uric acid; and weak negative correlation between Ca cor. values and HDL concentration: Ca cor./HDL (r=-0.141, p<0.001); Ca cor./TC (r=0.137; p<0.001); Ca cor./uric acid (r=0.210; p<0.001). PTH level had a weak negative correlation with HDL concentrations (r=-0.148; p<0.001). Expectedly, eGFR values were weakly negatively correlated with TC (r=-0.143; p<0.001), uric acid levels (r=-0.221; p<0.001), fasting glycaemia (r=-0.163; p<0.001), as well as with LV mass index (r=-0,206; p<0.001) and IST (r=-0,237;p<0.001).

### 3.6 Cardiovascular therapy and AH in patients with PHPT

We also analyzed a range of cardiovascular medications at the time of admission and at discharge. The evaluation was performed by class of medications without regard to specific substances and dosages. The results are summarized in **Table 6**.

**Table 6:** Cardiovascular therapy in patients on admission and at discharge from hospital.

Drug group	№ of patients at admission (n (%), N = 838)	№ of patients at discharge (n (%), N = 838)
Beta-blockers	250 (29.8%)	277 (33.1%)
ACEI	158 (18.9%)	155 (18.5%)
ARB	204 (24.3%)	248 (29.6%)
Dihydropyridine CCB	147 (17.5%)	210 (25.1%)
Non-dihydropyridine CCB	6 (0.7%)	1 (0.1%)
Thiazide/thiazide-like diuretics	114 (13.6%)	79 (9.4%)
Loop diuretics	23 (2.7%)	31 (3.7%)
Aldosterone antagonists	28 (3.3%)	25 (3.0%)
Carboanhydrase inhibitor	2 (0.2%)	2 (0.2%)
Alpha-adrenergic agonists	2 (0.2%)	3 (0.4%)
Imidazoline receptor agonists	23 (2.7%)	24 (2.9%)

ACEI, angiotensin-converting-enzyme inhibitors; ARB, aldosterone receptor blockers; CCB, calcium channel blockers.

Patients who received ACEI/ARB had lower eGFR than patients with other therapy (79 [66;88] vs. 89 [78;99] ml/min/1.73m^2^, p<0.001, U-test), lower osteocalcin (41 [26;66] vs. 47 [31;79] ng/ml, p=0.003, U-test) and CTX (0,8 [0,5;1,3] vs. 1,0 [0,6;1,4] ng/ml, p=0.003, U-test) concentrations. They did not differ in calcemia, phosphatemia and serum PTH. Patients who received CCBs also had lower eGFR than patients with other therapy, including ACEI/ARB (78 [63;89] vs. 86 [75;96] ml/min/1.73m^2^, p<0,001, U-test), and didn’t differ in calcemia, phosphatemia, bone resorption markers and serum PTH levels.

Patients with verified AH (n = 587) were selected for further analysis. Of all patients with AH, 90 (15.3%) had stage I, 306 (52.1%) had stage II, 152 (25.9%) had stage III of hypertension. IHD was diagnosed in 70 (11.9%) patients, 51 (72.8%) of whom had effort angina, and 16 (22.8%) had postinfarction cardiosclerosis. 36 (6.1%) patients with AH had a history of acute cerebrovascular events, 58 (9.8%) patients had CHF of various stages. According to echocardiography (data available for 280 patients), interventricular septum thickness was 11 [9; 12] mm and the posterior wall of the left ventricle was 10 [9; 11] mm.

For further analysis, these patients were divided into four subgroups according to the stage of AH: subgroup AH-I had stage I, AH-II- stage II, AH-III - stage III of AH. These subgroups were compared for parameters of mineral metabolism and incidence of classical complications of PHPT. The results are shown in **Table 7**. The subgroups differed in age (group AH-I patients were significantly younger) and in BMI (it was lower in group AH-I patients). More severe AH stage was associated with lower eGFR and higher fasting glucose levels. There were no differences between the subgroups in terms of mineral metabolism parameters and PHPT “classic” complications. As expected, patients with hypertension were older, had a higher BMI, higher glycemia, uric acid and triglyceride concentrations, and lower GFR and HDL concentration than patients without CVDs.

**Table 7:** State of mineral metabolism in patients with different stages of AH.

Indicator	Reference range	Patients without CVDs (n=226)		subgroup AH-I (n = 90)		subgroup AH-II (n = 306)		subgroup AH-III (n = 152)		р	р, *post-hoc* analysis
		*0*		*1*		*2*		*3*			
		n	Ме [Q1; Q3]	n	Ме [Q1; Q3]	n	Ме [Q1; Q3]	n	Ме [Q1; Q3]		
Sex, male	*–*	226	15 (7%)	90	9 (10%)	306	16 (5%)	152	10 (7%)	0.449^2^	*–*
Age, years	–	226	51 [39;58]	90	58 [53;64]	306	63 [57;69]	152	65 [58;71]	**<0.001 ^1^**	p_0-1_< 0.001 p_0-2_< 0.001 p_0-3_< 0.001 p_1-2_ < 0.001 p_1-3_ < 0.001 p_2-3 _= 0.043
BMI, kg/m^2^	20–25	226	25.7 [23.4;29.0]	90	27 [25; 30]	306	29 [27;4]	152	30 [26;36]	**<0.001 ^1^**	p_0-1 _= 0.102 p_0-2_< 0.001 p_0-3_< 0.001 p_1-2_ < 0.001 p_1-3_ < 0.001 p_2-3 _= 1.000
Са ionized, mmol/l	1.03–1.29	122	1.29 [1.25;1.37]	52	1.30 [1.24;1.37]	178	1.29 [1.24;1.35]	95	1.30 [1.26;1.36]	0.609 ^1^	–
Са corr., mmol/l	2.15–2.55	226	2.65 [2.56;2.81]	89	2.68 [2.56;2.80]	305	2.67 [2.57;2.84]	151	2.69 [2.59;2.82]	0.520 ^1^	–
Phosphorus, mmol/l	0.74–1.52	218	0.86 [0.75;0.97]	88	0.86 [0.76;0.95]	300	0.88 [0.78;0.96]	145	0.88 [0.77;0.98]	0.321 ^1^	–
eGFR by CKD-EPI, ml/min/1,73m^2^	–	224	93 [84;1.3]	89	85 [74;96]	306	81 [67;90]	151	79 [66;89]	**<0.001 ^1^**	p_0-1_< 0.001 p_0-2_< 0.001 p_0-3_< 0.001 p_1-2 _= 0.005 p_1-3 _= 0.001 p_2-3 _= 0.945
25 (ОН) D, ng/ml	30–100	108	22 [15;32]	55	24 [15;34]	143	23 [16;30]	71	26 [19;36]	0.125 ^1^	-
PTH, pg/ml	15–65	226	124 [98;205]	90	130 [110;169]	306	136 [102;2017]	152	133 [101;220]	0.535 ^1^	–
ALP, u/l	40–150	193	79 [62;107]	79	91 [68;111]	287	91 [70;117]	131	86 [70;118]	0.024 ^1^	-
Osteocalcin, ng/ml	11–43	162	43 [30;68]	72	45 [31;73]	214	45 [28;77]	110	41 [25;68]	0.572 ^1^	-
CTX, ng/ml	0.3–0.57	160	0.91 [0.57;1.37]	72	0.98 [0.63;1.30]	214	0.87 [0.50;1.36]	111	0.71 [0.38;1.26]	0.050 ^1^	–
Fasting glucose, mmol/l	3.1–6.1	209	5.06 [4.71;5.38]	87	5.13 [4.78;5.48]	278	5.30 [4.96;5.73]	138	5.41 [4.99;5.91]	**<0.001 ^1^**	p_0-1 _= 0.224 p_0-2_< 0.001 p_0-3_< 0.001 p_1-2 _= 0.012 p_1-3_ < 0,001 p_2-3 _= 0,443
TC, mmol/l	3.3–5.2	223	5.46 [4.89;6.31]	90	5.7 [5.0;6.3]	305	5.4 [4.7;6.3]	148	5.34 [4.23;6.31]	0.086 ^1^	–
HDL, mmol/l	1.1–3	220	3.5 [2.8;4.3]	85	1.4 [1.2;1.6]	283	1.3 [1.1;1.5]	139	1.3 [1.1;1.5]	**<0.001 ^1^**	p_0-1 _= 0.596 p_0-2_< 0.001 p_0-3_< 0.001 p_1-2 _= 0.017 p_1-3 _= 0.005 p_2-3 _= 0.436
LDL, mmol/l	1.15–2.6	209	1.47 [1.17;1.72]	90	3.7 [3.0;4.4]	302	3.5 [2.7;4.3]	149	3.4 [2.3;4.3]	**<**0.051^1^	–
Triglycerides, mmol/l	0.1–1.7	218	1.17 [0.87;1.65]	87	1.1 [0.9;1.5]	301	1.4 [1.0;0.9]	149	1.3 [1.0;1.9]	**<0.001 ^1^**	p_0-1 _= 0.869 p_0-2_< 0.001 p_0-3 _= 0.008 p_1-2 _= 0.003 p_1-3 _= 0.021 p_2-3 _= 0.659
Uric acid, mmol/l	142–339	167	307 [270;345]	67	324 [284;367]	252	351 [295;417]	124	363 [307;433]	**<0.001 ^1^**	p_0-1 _= 0.044 p_0-2_< 0.001 p_0-3_< 0.001 p_1-2 _= 0.026 p_1-3 _= 0.003 p_2-3 _= 0.205
Bone complications		226	124 (54%)	90	56 (62%)	306	206 (67%)	152	107 (70%)	0.006^2^	–
Visceral complications		226	138 (61%)	90	54 (60%)	306	192 (63%)	152	101 (66%)	0.693^2^	–

^1^ – Kruskal–Wallis test; ^2^ – χ^2^ test; р_0 _= 0.05/20 = 0.003 (Bonferroni correction).

Bold values mean statistic significance of the p-value.

## 4 Discussion

Our study included more than 800 PHPT patients. Among them, the rate of “classic” PHPT complications - bone and visceral manifestations - was up to 70% and 83%, respectively. Thus, the incidence of bone complications of PHPT among our patients was higher than previously thought (9, 10). Visceral complications of PHPT in our study were also more frequent, than in literature data (9–12). There may be several reasons for these peculiarities. Firstly, this study included patients of a federal specialized medical facility, where patients with the most severe and/or atypical forms of the disease are referred to, which biases the sample. Secondly, the data in the nationwide studies can be incomplete both due to insufficient examination of patients. At the same time, in our work, renal ultrasound/MSCT data were available for the vast majority of patients, which allows us to consider the data obtained as representative for patients admitted with PHPT. In contrast, esophagogastroduodenoscopy was only performed when indicated, which necessitates further specification of the incidence of gastrointestinal erosions and ulcers in PHPT. However, the patients in other studies are not age-matched to the sample under study, so it is inappropriate to make direct comparisons.

In the different regions of the Russian Federation obesity (BMI ≥30 kg/m^2^) varies from 22.5% to 44.5% (13). This rate is relatively high; however, it is comparable with data in other developed countries (14). The incidence of obesity among patients with PHPT in our study reached 24–36% depending on the age group, which is generally consistent with the Russian population data. At the same time, people with PHPT are believed to be more often obese than the general population. For example, Bolland et al. in the meta-analysis including 617 patients with PHPT and 1248 healthy volunteers demonstrated a direct relationship between PHPT and BMI, while body weight did not correlate with vitamin D concentrations and severity of hypercalcemia (15). However, increased body weight was probably a result of hypodynamia caused by bone lesions (the study did not assess patients’ motor activity). In another large study (n = 9114), obesity in PHPT was associated with a higher risk of the post-surgical relapse. The annual rate of reoperation was 3.6%-4.8%, and 48.5% of patients undergoing re-surgery were obese compared to 40% receiving initial parathyroidectomy (p = 0.009). The likelihood of reoperation rises with increasing BMI from 2.1% for underweight to 5.0% for obesity (p = 0.009) (16). Excess adipose tissue is thought to complicate surgical access to the parathyroid glands, making radical parathyroidectomy difficult.

There is evidence, that dyslipidemia as a component of the metabolic syndrome is more common in PHPT than in the general population. Thus, antihyperlipidemic medications were more frequently prescribed for patients with PHPT compared to age- and sex-matched controls (19.1% and 5.9%, respectively; p = 0.02). Interestingly, hypertriglyceridemia and decreased serum HDL level were more typical for mild PHPT, while high LDL levels have been noted for symptomatic and asymptomatic patients (17). In our study, we observed frequent dyslipidemia: increased LDL >3.0 mmol/l was found in 62-67%, hypertriglyceridemia in 26-28%, and hyperuricemia in 39-49% of patients. This is consistent with epidemiological data on lipid and purine metabolism pathology for russian population: 59.7 ± 0.34% of the population had elevated LDL, 19.5 ± 0.28% had hypertriglyceridemia (18), and 16.8% had hyperuricemia (19). At the same time, hyperuricemia among our patients seems to be more than in the general population. In addition, weak positive correlations between serum calcium and uric acid levels were revealed. Similar data were demonstrated in a systematic review, in which patients with PHPT had a significantly higher level of serum uric acid than individuals without hyperparathyroidism with the pooled mean difference of 65.00 μmol/L (95% CI 37.74-92.25) (20).

There are some links between PHPT and carbohydrate metabolism disorders (mainly diabetes mellitus), but the exact mechanism of this association is still unclear. Calcium can suppress the insulin binding to its receptor: this leads to a positive correlation between serum calcium, fasting glycemia and insulin resistance in patients with PHPT (n = 1182) (21).

According to the literature data, 8–22% of patients with PHPT suffer from diabetes, and the incidence of PHPT in diabetes is close to 1%. IFG/IGT is also more common in PHPT than in the general population (22). In the Russian population, the overall prevalence of DM is about 5.4%, and it reaches 14.1% in women older than 60 years of age (23). In other populations the prevalence of DM may reach 8.5 - 11.7% (24, 25). In the present study, the prevalence of DM among all PHPT patients was 12.4%, which is higher than in Russian population. It should be noted, however, that the sample studied was composed mainly of patients in the older age group, and that the majority of the patients were women. This fact predominantly illustrates increased diabetes prevalence with age; however, it does not allow to rule out the additional unfavorable effect of PHPT on carbohydrate metabolism. The incidence of IFG/IGT for all groups (2.0% in group A and 7.3% in group B) in this study was lower than in the general russian population (19.3%) (23), despite the significant number of obese patients.

AH is one of the most frequent CVDs, affecting up to 29% of the population. Its prevalence increases with age and reaches 7.3% at the age of 18–39 years; 32.2% - for 40–59 years; 64.9% at the age over 60 years (26). Worldwide, 40% to 60% of PHPT patients have AH (5, 27). We have shown that the incidence of AH in PHPT is higher than in the general population, and significantly increases with age, reaching 79.1% in patients over 50 years old. In our work, with age-associated increase of AH frequency, the probability of LVH also increases. These deviations are consistent with the ideas about the pathogenesis of AH in total and with the data indicating a higher prevalence of LVH in patients with PHPT than in a comparable control group (28, 29).

The incidence of IHD is estimated to be 6.3% among men 40–59 years old and 5.6% among women; by 60–79 years old, IHD affects up to 19.9% and 9.7% of men and women, respectively (30). Comparative studies have previously shown a higher incidence of IHD in PHPT than in the general population (6.95% vs 3.7%) (27). In our study, 10.8% of older patients had a history of IHD which is consistent with the study above.

Various cardiac rhythm disturbances were common in our cohort reaching 11.8% in the elder group. This is comparable with previously published clinical data and some cases (31). For example, Kelesoglu et al. showed that the risk of atrial fibrillation in PHPT is higher than in people without calcium metabolism disorders (32). Nevertheless, data on the risk of arrhythmia in PHPT patients is still limited.

The high incidence of atherosclerosis of BCA and lower limb arteries in our study (in the older age group up to 84% and 48.7%, respectively) deserves special attention. This can be explained by high pretest probability of atherosclerosis detection, because the ultrasound scan was performed only when indicated, and not as screening. Moreover, Streeten et al. found no differences between the incidence of coronary artery calcification in PHPT and in healthy subjects (33), and Ogard et al. did not prove a link between serum concentrations of atherosclerosis markers (brain natriuretic peptide, CRP and tumor necrosis factor-ɑ) and PTH or calcium levels. Moreover, no decrease in NT-proBNP, markers of inflammation or blood pressure was observed after parathyroidectomy (34). However, even in the asymptomatic PHPT, parathyroidectomy is associated with a thinner intima-media complex, than conservative treatment (35); and an endothelial dysfunction in PHPT can develop without the signs of subclinical atherosclerosis (36, 37).

The main factors increasing the likelihood of CVD in a patient with PHPT are age, GFR, and excess body weight. In particular, BMI over 22.86 kg/m^2^ in men and 24.02 kg/m^2^ in women is an independent risk factor for AH (38). It is associated with both increased amount of epicardial adipose tissue, chronic inflammation, obstructive sleep apnea syndrome, as well as carbohydrate and lipid metabolism disorders and atherosclerosis caused by obesity (39). In turn, central obesity alone significantly increases the prevalence of atherosclerosis (e.g., 52% vs. 28%, p = 0.02) according to Salari et al. (40). BMI positively correlates with triglyceride (41) and uric acid (42) concentrations. Age is a well-studied risk factor for AH (43) and atherosclerosis (44) too. It is also known that decreased renal filtration function increases the probability of AH (45), atherosclerosis (46), hypertriglyceridemia (47) and hyperuricemia (48). No direct relationship between mineral metabolism and CVD risk in patients with PHPT was found in present study, which may indicate more complex pathogenic associations between these conditions.

It is noteworthy that only the lipid profile and blood uric acid were directly linked to parameters of calcium metabolism. Hyperuricemia is typical for PHPT but the reasons for this relationship are still debated. One of the most likely mechanisms is the combination of decreased renal clearance of urate with an increased nitrogen-containing bases in blood along with more active bone metabolism (which is established by alkaline phosphate concentration). Our results, therefore, are consistent with the existing data (20, 49). Dyslipidemia, in particular hypertriglyceridemia, is also observed more frequently in PHPT than in the general population (50). Presumably this is due to PTH-mediated inhibition of lipoprotein lipase (51, 52). This does not contradict our results: the primary pathogenetic factor of hypertriglyceridemia could be increased PTH, and the association with hypercalcemia in this case is indirect. At the same time, the diagnostic value of the associations we found was low. This may be a consequence of the study design, its power, or the lack of hypothetical relationships described above. Further research is needed to interpret these results and develop practical algorithms.

The relationship between cardiotropic therapy taken by patients and mineral metabolism parameters is challenging. A connection between PHPT and AH has been previously confirmed by several studies (53–56), and the impact of various components of the renin-angiotensin-aldosterone system (RAAS) on Ca-P metabolism is also well known. For example, one of the largest studies (n = 5668) of patients with PHPT confirmed a significant positive relationship between aldosterone and PTH. Moreover, PTH levels increased incrementally from normotensive to hypertensive patients receiving RAAS inhibitors, hypertensive patients without therapy, and hypertensive patients receiving other antihypertensive medications. Taking any RAAS inhibitors was an independent predictor of lower blood PTH concentrations; other antihypertensive medications did not show the similar effect (57). According to The Multi-Ethnic Study of Atherosclerosis, the thiazide therapy in patients without PHPT and with preserved GFR (n = 1888) was associated with lower PTH values (due to its hypocalciuric effect), while loop diuretics and dihydropyridine CCBs - with higher ones (58). Thus, benefits of some antihypertensive agents for patients with PHPT may be expected, but further research is required.

In our study, the control of AH predictably depended on the renal filtration function (59). At the same time, there was no difference in mineral metabolism parameters between patients with different stages of AH. A prospective study on patients with PHPT without AH and with AH without history of antihypertensive therapy is necessary to clarify the course of AH in PHPT and effects of different classes of antihypertensive drugs on Ca-P metabolism. However, AH can be the major (sometimes the only) manifestation of PHPT (60), which cannot be ignored by deciding on surgery.

Patients with PHPT most often received beta-blockers (33.1%), ARBs (29.6%), and dihydropyridine CCBs (25.1%). This distinguishes our group from the general population with AH, who takes ACEIs more frequently (61). The fact that ARBs was preferred over ACEIs may be indirectly related to the described effect of PTH on aldosterone synthesis (57) and, therefore, the better effect of ARB in blood pressure normalization. Our patients received thiazide/thiazide-like diuretics relatively frequently - in 9.4% of cases, which exceeds the prescription rate of these drugs among all patients with AH (62). This choice of drug is questionable due to the mechanism of their action. Thiazide diuretics exert their effect via blockage of the sodium-chloride (Na/Cl) channel in the proximal segment of the distal convoluted tubule. At the same time, Na/Cl channel blockage increases the flow of ions through the Na/Ca channel, resulting in increased calcium reabsorption into the interstitium in exchange for Na return. However, in some PHPT cases it can be useful: for example, in moderate hypercalcemia and severe hypercalciuria. Small doses of hydrochlorothiazide (12.5-25 mg/day) can prevent nephrocalcinosis/nephrolithiasis and do not lead to significant increase in blood calcium levels (63).

## 5 Study limitations

The study was conducted in a federal medical institution, which predominantly admits patients with a complex and/or atypical course of the disease, which may have led to a bias in the resulting sample. At the same time, the clinical and demographic characteristics of the studied group are comparable to those of the Russian Registry. The second significant limitation of this work is the absence of the group of healthy controls for comparison; therefore, the data obtained can be compared only against those from previously published works. The lack of dynamic follow-up of patients after parathyroidectomy also limits the interpretation of the data obtained.

A number of studied parameters are characterized by incomplete information (some studies performed in patients only when indicated).

Part of the group was enrolled during the COVID pandemic. This may have distorted the sample because the COVID restrictions had an impact on medical care organization.

The study did not take into account the doses of antihypertensive drugs taken by patients and did not conduct daily BP monitoring, which limits the possible depth of interpretation of the revealed relationships.

## 6 Conclusion

The present study demonstrated a high incidence of some CVD, as well as disorders of lipid, carbohydrate and purine metabolism in patients with PHPT. In groups of different age, AH was found in 28.9-79.1%, IHD - in 0.7-9.8%, CHF - in 0-8.4%, type 2 DM - in 2.6-14.4%, prediabetes - in 2.0-7.3%, obesity - in 24.2-35.9% of cases. We did not find strong correlations between the main parameters of mineral metabolism and CVD and MD. However, the obtained data determine the need for further studies to clarify the role of PHPT in the development of CVD and MD, as well as to create new approaches to their treatment and effective prevention.

## Data availability statement

The raw data supporting the conclusions of this article will be made available by the authors, without undue reservation.

## Ethics statement

The studies involving humans were approved by Ethics Committee of the Endocrinology Research Centre. The studies were conducted in accordance with the local legislation and institutional requirements. The participants provided their written informed consent to participate in this study.

## Author contributions

ED: Conceptualization, Supervision, Writing – review & editing. AG: Data curation, Formal Analysis, Investigation, Methodology, Writing – original draft, Writing – review & editing. EB: Conceptualization, Data curation, Formal Analysis, Investigation, Methodology, Writing – original draft, Writing – review & editing. AKE: Conceptualization, Methodology, Project administration, Supervision, Writing – review & editing. ARE: Data curation, Formal Analysis, Software, Writing – review & editing. RS: Data curation, Investigation, Methodology, Writing – review & editing. EK: Conceptualization, Formal Analysis, Supervision, Writing – review & editing. IM: Investigation, Writing – review & editing. NM: Conceptualization, Project administration, Supervision, Writing – review & editing.
